# *Artocarpus tonkinensis* Extract Inhibits LPS-Triggered Inflammation Markers and Suppresses RANKL-Induced Osteoclastogenesis in RAW264.7

**DOI:** 10.3389/fphar.2020.593829

**Published:** 2021-01-22

**Authors:** Elena Orecchini, Giada Mondanelli, Ciriana Orabona, Claudia Volpi, Sabrina Adorisio, Mario Calvitti, Trinh Thi Thuy, Domenico V. Delfino, Maria Laura Belladonna

**Affiliations:** ^1^ Department of Medicine and Surgery, University of Perugia, Perugia, Italy; ^2^ Department of Medicine and Surgery, Foligno Nursing School, University of Perugia, Perugia, Italy; ^3^ Institute of Chemistry, Vietnam Academy of Science and Technology, Hanoi, Vietnam

**Keywords:** *Artocarpus tonkinensis*, osteoclastogenesis, rheumatoid arthritis, hmong ethnic minority, vietnam traditional medicine

## Abstract

*Artocarpus tonkinensis* (*At*) leaf decoction, a traditional remedy prepared in North *Vietnam* by the Hmong ethnic group, is a tea extract rich in bioactive compounds that may have therapeutic effects in arthritis and backache. Indeed, it has been demonstrated that *At* is able to inhibit Th17 lymphocytes development and to protect mice in an experimental model of collagen-induced arthritis. By resorting to macrophage *in vitro* models of inflammation and osteoclastogenesis, we showed that *At* extract significantly reduced nitric oxide synthase 2 (NOS2) activity and IL-6 production by RAW 264.7 murine cells. Moreover, *At* demonstrated an anti-osteoclastogenic effect, as revealed by complete inhibition of TRAP-positive osteoclast formation and decreased expression of key osteoclast-related genes. This *At* activity likely relies on the inhibition of RANK downstream signaling pathway, as the activation of non-receptor tyrosine kinase Src is reduced upon RANKL-*At* exposure. Protective effect of *At* against bone loss was also enlightened *in vivo* by collagen-induced arthritis (CIA) experiment demonstrating that, although paw edema was only weakly opposed by drinking *At* decoction, bone and cartilage were well preserved in CIA+*At* mice and joint tissue expressed decreased levels of osteoclast marker genes respect to CIA control group. Maesopsin 4-O-β-D-glucoside (i.e., TAT-2, one of the main decoction bioactive components) was capable to contrast NOS2 activity, IL-6 expression and osteoclast formation, too, albeit to a lesser extent when compared to *At* decoction. Overall, this study enlightens another *At* cell target, macrophages, beside Th17 lymphocytes, and suggests that the anti-arthritic beneficial effects of *At* decoction largely derives from its ability to counteract not only inflammation, but also osteoclastogenesis.

## Introduction

*Artocarpus tonkinensis* (A.Chev. ex Gagnep) is a tree belonging to the Moraceae family. Found in many tropical regions of Asia and Africa, it abundantly grows in northern Vietnam, where it is used as herbal medicine by the Hmong ethnic minority to treat arthritis and backache (Adorisio et al., 2016; Adorisio et al., 2019). Medicinal properties of leaf and root extracts from *A. tonkinensis* have long been known and they are recorded in the textbooks of traditional Vietnamese and Chinese medicine. In particular, *A. tonkinensis* dried leaf decoction and its major active compounds auronol glycosides maesopsin 4-O-β-D-glucoside (TAT-2) and alphitonin-4-O-β-D-glucoside (TAT-6) were studied for their immunosuppressive activity in autoimmune diseases (Thuy et al., 2004; Thuy et al., 2017) and for anti-proliferative effect in anti-cancer trials (Thuy et al., 2016). Vietnamese folk medicine practice to assume *A. tonkinensis* decoction against rheumatoid arthritis (RA) symptoms and several studies on experimental models of collagen-induced arthritis (CIA) (Ngoc et al., 2005; Dang et al., 2009; Adorisio et al., 2019) support the awareness of *A. tonkinensis* efficacy to contrast chronic inflammation, and destruction of bone and joints characterizing RA pathological picture.

RA is an autoimmune human disease initiated by still unknown processes. It displays an intense activation of innate immune system, including monocyte/macrophage cells, arachidonic acid metabolites and inflammatory cytokines, and triggering a severe inflammatory state responsible for joint pain, swelling and destruction of cartilage and bone. T cells accumulating in the inflamed synovium likely drive a T cell dependent reaction to a still unknown antigen, while activated cells of macrophage and fibroblast origin dominate the joint destructive process (Scherer et al., 2020). Chronically-activated macrophages maintain a pro-inflammatory phenotype producing large amounts of inducible nitric oxide synthase (NOS-2), cytokines (among them, TNFα, IL-1β and IL-6) and chemokines, triggering neighboring fibroblasts to proliferate and produce tissue degrading enzymes, receptor activator of NF-κB ligand (RANKL) and macrophage colony-stimulating factor 1 (M-CSF). Overproduction of RANKL (the ligand of RANK transducing the osteoclastogenic signal) and M-CSF (a hematopoietic growth factor), highly promotes the formation of osteoclasts. These multinucleated cells, derived from the fusion of hematopoietic mononuclear precursors of monocytic lineage, are coordinated with osteoblasts for physiological bone remodeling, but in RA, the unbalanced differentiation of them is responsible for pathologic cartilage destruction and bone resorption (Siouti and Andreakos, 2019).

Previous studies by Adorisio et al. (Adorisio et al., 2019) demonstrated the immunoregulatory activity of *A. tonkinensis* water extract in CIA mouse model. *A. tonkinensis* decoction limited CIA progression when it was administered after arthritis development and prevented CIA if it was given before appearance of clinical signs. The study, focused on lymphocyte subpopulation involvement, indicated Th17 as the T cell subset mainly inhibited by *A. tonkinensis* bioactive components, both *in vivo* (reduced Th17 markers in joints of decoction-treated CIA mice) and *in vitro* (reduced Th17 polarization from CD4^+^ splenic T cells in the presence of *A. tonkinensis* water extract). Here, we investigated whether also macrophage cell population might be targeted by *A. tonkinensis* decoction and by its major component TAT-2, assessing if they could interfere in macrophage acquisition of a pro-inflammatory phenotype, and decrease formation of osteoclasts, key cells in the RA pathologic joint damage. Using the established inflammation *in vitro* model of RAW264.7 macrophage cell line conditioned with LPS (Fan et al., 2020), we investigated the effects on nitric oxide (NO) and IL-6 production. Moreover, inducing osteoclastogenesis in RAW264.7 by RANKL protein (Kim et al., 2019), we evaluated the inhibition of osteoclast differentiation process and the decrease of its markers, also exploring the interference at RANK signaling level.

## Materials and Methods

### Reagents

LPS from *E. Coli* serotype 055:B5 and 3-(4,5-dimethylthiazol-2-yl)-2,5-diphenyltetrazolium bromide (MTT) were purchased from Sigma Aldrich (St. Louis, MO, United States). As TRAP staining reagent, Acid phosphatase Kit 387-A was used (Sigma-Aldrich, St. Louis, MO, United States). TRAP reaction buffer was prepared as described by the manufacturer’s instructions (Sigma Aldrich, St. Louis, MO, United States). Recombinant murine RANKL was from Peprotech (PeproTech EC, Ltd., London, United Kingdom). pSrc/Src ratio was assessed by immunoblot with rabbit Phospho-Src Family (Tyr416) Antibody #2101 (Cell Signaling Technology, Danvers, MA, USA), recognizing the phosphorylation at tyrosine 424 in murine c-Src, followed by the detection of total Src by rabbit monoclonal antibody 36D10 (Cell Signaling Technology, Danvers, MA, United States), as previously shown (Manni et al., 2020).

### *A. tonkiniensis* Water Extract

Leaves of *At* were harvested in Hanoi, Vietnam in October 2015, and the voucher species (No. AT-2015) was deposited in the Institute of Chemistry, Vietnam Academy of Science and Technology. Briefly, freshly harvested leaves were air-dried for 2 days and oven-dried at 40°–50°C in a forced-air oven. To obtain the decoction, 5 g of crushed dried leaves were boiled 3 times in 100 ml total volume of RPMI-1640 medium and total water extract *At* was filtered through a 0.22 μm syringe filter before supplementation with FBS 10% and other culture additive. Chemical analysis of *At* water extract was previously published in our study by Adorisio et al., 2017. *At* water extract (decoction) we used in the present work belonged to the very same batch of *At* (No. AT-2015) already prepared for such prior study, where chemical composition was determined by HPLC analysis Adorisio et al., 2017 and revealed the presence of seven compounds qualitatively and quantitatively quite constant in independent experiments. *At* water extract fingerprint included catechin, alphitonin-4-O-β-D-glucoside (TAT-6), maesopsin-4-O-β-D-glucoside (TAT-2) (Supplementary Figure S1), quercetin-3-β-D-glucoside, kaempferol-3-O-glucoside, quercetin, kaempferol. Concentrations of the most abundant compounds, TAT-6 and TAT-2, were 142.8 µg/ml and 122.5 µg/ml, respectively (Adorisio et al., 2019). The very same batch of *At* (No. AT-2015) was used in all the experiments of the present work. The TAT-2 compound was isolated as previously described (Adorisio et al., 2019).

### Cell Culture

The murine macrophage cell line RAW 264.7 was obtained from the American Type Culture Collection (ATCC, Manassas, VA, United States). Cells were cultured according to standard procedures in RPMI-1640 medium, supplemented with 10% heat-inactivated Fetal Bovine Serum (FBS), 2 mM of L-glutamine and antibiotics (100 U/ml penicillin, 100 μg/ml streptomycin). The cultures were never allowed to become confluent and medium was changed every 3 days. Incubations were performed at 37°C in 5% CO_2_ atmosphere and humidified air. In the experiments, RAW 264.7 cells were seeded into wells of culture plates and incubated overnight prior to treatment. Supernatants were then replaced with medium containing scalar amounts of *At* (0.2–12.8 mg/ml), or scalar concentrations of TAT-2 (1, 10, or 100 μg/ml), in the presence of LPS 50 ng/ml (proinflammatory model) or RANKL 100 ng/ml (osteoclastogenic model). In MTT and nitric oxide colorimetric assays, after incubation with proper stimuli, cells were washed before addition of specific colorimetric reagents and absorbance reading. Analysis of c-Src phosphorylation was performed on either undifferentiated or RANKL-differentiated RAW 264.7; cells were pre-treated with *At* (3.2 mg/ml) or TAT-2 (100 μg/ml) for 1 h before the stimulation with RANKL (100 ng/ml) for the indicated time.

### Cell Viability Assay

Cell viability was measured by MTT assay. RAW 264.7 cells (2 × 10^4^ and 1 × 10^4^ cells/cm^2^ for LPS-and RANKL-stimulated samples, respectively) were seeded into flat bottom 96-well plates. After incubation with stimuli (*At* extract 0.2–12.8 mg/ml, or TAT-2 1, 10 and 100 μg/ml), in the presence of LPS (50 ng/ml for 24 h) or RANKL (100 ng/ml for 5 days), adherent cells were washed once to remove any residual *At* brown extract, added with 100 μl of medium containing MTT 10 μl (5 μg/ml) per well and incubated for 4 h at 37°C. Then, 100 μl of solubilization buffer (SDS 10% in HCl 0.01 N) were added to each well, plate was incubated at 37°C overnight and absorbance at 570 nm was measured using an UV/visible spectrophotometer (TECAN, Thermo Fisher Scientific, Waltham, MA, United States). The assay was performed in triplicate for each concentration.

### NO Assay

RAW 264.7 cells (1.5 × 10^5^ cells/well) were seeded into flat bottom 96-well plates and incubated overnight prior to the experiment. Supernatants were removed and cells were cultured with scalar concentrations of *At* (0.2–6.4 mg/ml) or TAT-2 (1, 10 and 100 μg/ml) in the presence of LPS 50 ng/ml for 24 h. After 24 h incubation, adherent cells were washed to remove any residual *At* brown extract and incubation was resumed in fresh culture medium for additional 24 h. To assay the total production of NO, 50 μl of each culture supernatant and 100 μl of Griess reagent (1 part of 1% naphthylethylenediamine dihydrochloride in distilled water plus one part of 1% sulfanilamide in 5% concentrate phosphoric acid) were incubated for 7 min, light protected and at room temperature. Samples absorbance was read at 530 nm using a spectrophotometer (TECAN, Thermo Fisher Scientific, Waltham, MA, United States) and nitrite concentration was quantified by comparison with a sodium nitrite standard curve. The assay was performed in triplicate for each concentration.

### TRAP Staining

TRAP cell staining was evaluated in RANKL-induced differentiation cultures of RAW 264.7. Three × 10^3^ cells per well (1 × 10^4^ cells/cm^2^) were seeded into flat bottom 96-well plates for overnight adherence. Culture medium was then removed and cells were stimulated for 5 days with different amounts of *At* extract or TAT-2, in the presence of RANKL 100 ng/ml to induce differentiation of RAW 264.7 macrophages into osteoclasts. Medium containing stimuli was changed every two days. After 5 days, cells were stained by TRAP reagent, according to the manufacturer’s protocol. Under a bright-field light microscope (EVOS M5000, Thermo Fisher Scientific, Waltham, MA, United States) TRAP-positive multinucleated cells having three or more nuclei were considered as osteoclasts, and their numbers counted in randomly selected visual fields in different areas of each well.

### Real-Time PCR and IL-6 Measurement

Total RNA was extracted from cells by TRIzol (Invitrogen, Carlsbad, CA, USA) and reverse transcribed to cDNA with QuantiTect Reverse Transcription Kit (Qiagen, Hilden, Germania). Real-time PCR was performed using SYBR Green detection and specific primers listed in Table 1. Values were calculated as the ratio of the specific gene to *Gapdh* expression, as determined by the relative quantification method (ΔΔCT; means ± SD of triplicate determination) (Mondanelli et al., 2020). IL-6 occurrence in culture supernatants was detected using mouse IL-6 uncoated ELISA Kit (Invitrogen, Carlsbad, CA, United States) as previously described (Mondanelli et al., 2017a) and according to manufacturer’s protocol. The average minimum detectable concentration of mouse IL-6 was 0.004 ng/ml.

**TABLE 1:** Primer sequences used in Real-time PCR.

Gene	Genbank accession number	Sequence (5′→3′)	Amplicon size
*Gapdh*	NM_001289726	CTG​CCC​AGA​ACA​TCA​TCC​CT	159 bp
		ACT​TGG​CAG​GTT​TCT​CCA​GG	
*Acp5*	NM_001102405	CTG​CCT​TGT​CAA​GAA​CTT​GC	168 bp
		ACC​TTT​CGT​TGA​TGT​CGC​AC	
*Calcr*	NM_007588	TCA​TCA​TCC​ACC​TGG​TTG​AG	275 bp
		CAC​AGC​CAT​GAC​AAT​CAG​AG	
*Mmp9*	NM_013599	GCT​GAC​TAC​GAT​AAG​GAC​GGC​A	114 bp
		GCG​GCC​CTC​AAA​GAT​GAA​CGG	
*Ctsk*	NM_007802	AGA​AGA​CTC​ACC​AGA​AGC​AG	98 bp
		CAG​GTT​ATG​GGC​AGA​GAT​TTG	

### Eliciting CIA in Mice

DBA/1J female mice, 8–12 weeks old, were supplied by Biogem SCARL (Ariano Irpino, Italy). CIA was elicited by intradermal immunization with 50 µL/mouse collagen type II (Sigma, C9301-5MG, Lot#016M4158V; St. Louis, MO, United States) emulsified in complete Freund’s adjuvant (Sigma, F5881-10ML, Lot#SLBQ1106V) on day 1 and in incomplete Freund’s adjuvant (Sigma, F5506-10ML, Lot#SLBL9742V) on day 31. Arthritis was induced in 10 mice (two groups of five mice each), while five mice composed the untreated negative control group. The injection has been performed at about 1.5 cm distal from the base of the tail, being careful to choose a tissue site and not a vessel. 50 μl of emulsion have been slowly injected intradermally into the tail while the mice were under gas anesthesia (2% isoflurane). Physical appearance, behavior and general and local clinical signs of the mice have been observed daily. To follow arthritis development, each paw has been evaluated and scored individually on a scale 0–4, with 0 indicating no inflammation and four indicating the most severe inflammation (Brand et al., 2007). Animal experiments were performed after approval by the ethical committee of Heath Ministery.

### Histological Assessment

One knee joint per mouse was removed, fixed in 10% (v/v) formalin for 24 h, decalcified in 5% (v/v) trichloroacetic acid for 7 days, dehydrated, embedded in paraffin, sectioned into 3- to 4-mm thick sections, and stained with hematoxylin and eosin.

### Statistical Analysis

All *in vitro* determinations are means ± SD from at least three independent experiments. Statistical significance was determined by the ANOVA one-way analysis (for different *At* or TAT-2 concentrations, treated vs untreated sample; for pSrc/Src at different time points, *At*+RANKL-vs RANKL-stimulated cells; for *in vivo* experiment at different time points, mean arthritic score of CIA vs Control and CIA+*At* vs CIA group). In real-time PCR analysis of mRNA relative expression in CIA+*At* vs CIA, and, at a unique TAT-2 dose, paired data (treated vs untreated sample) were evaluated by Student’s *t* test.

## Results

### Anti-Inflammatory Activity of *At* Extract on RAW264.7/LPS

**FIGURE 1:**
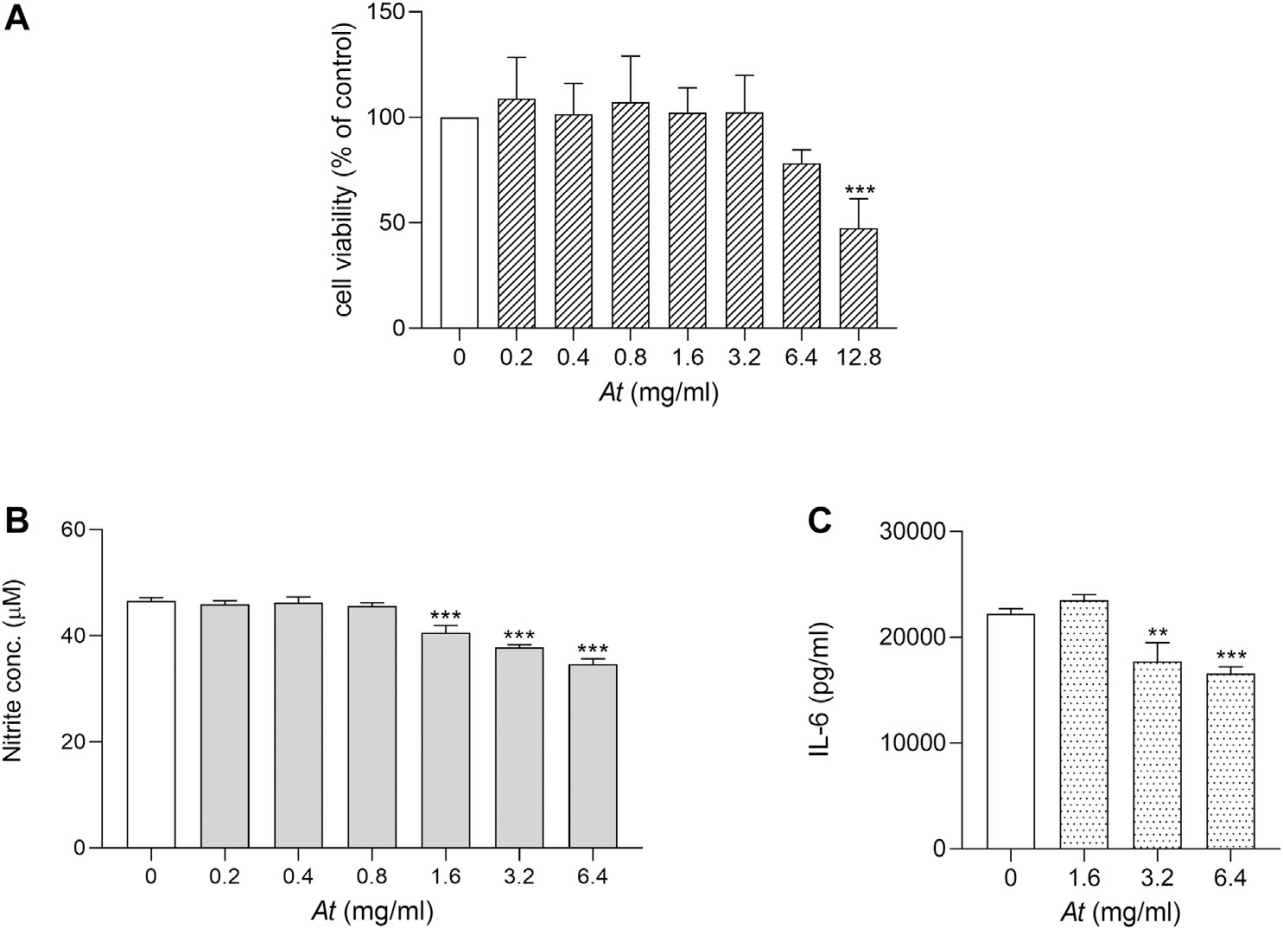
Viability and anti-inflammatory activity of *At* extract on LPS-activated RAW264.7 cells. LPS-activated cells were treated for 24 h with *At* extract at the indicated concentrations. **(A)** Cell viability was evaluated by MTT assay. The percentage of viable cells respect to LPS control was reported as the mean ± SD of three independent experiments, each conducted in triplicate. **(B)** NO release in the culture supernatant of LPS-activated RAW 264.7 cells was quantified as nitrite concentration. Results are shown as mean ± SD of three independent experiments, each conducted in triplicate. **(C)** IL-6 concentrations in culture supernatants of LPS-activated RAW 264.7 cells *in vitro* stimulated with the indicated concentrations of *At* extract were determined by ELISA test. Results are reported as mean ± SD of three independent experiments, each conducted in triplicate. **p < 0.01, ***p < 0.001, *At*-treated vs LPS-treated cells (one-way ANOVA test).

Our previous study, analyzing *in vitro* activity of *At* water extract on cell cultures, demonstrated *At* decoction efficacy in inhibiting Th17 differentiation and in protecting mice from autoimmune reactivity in a model of CIA (Adorisio et al., 2019). As macrophages play a key role in triggering inflammatory arthritis, LPS-stimulated RAW264.7 cell model was chosen to evaluate *At* anti-inflammatory effect on such cell type. We first verified whether *At* water extract negatively impacted cell viability in experimental culture conditions. Serial 2-fold dilutions of *At*, ranging 0.2–12.8 mg/ml, were used in the presence of LPS 50 ng/ml to evaluate RAW264.7 cell viability by MTT assay. After 24 h of incubation, *At*-treated samples and LPS control were similar in cell viability, but a slight decrease at 6.4 mg/ml and a significant reduction (by 47% ± 14%, *p* < 0.0001) was observed at the highest *At* concentration (i.e., 12.8 mg/ml) (Figure 1A). Thus, the 0.2–6.4 mg/ml concentration range, not affecting cell viability of LPS-cultured RAW 264.7 macrophages, was considered suitable for the subsequent evaluation of *At* activity.

To investigate the anti-inflammatory effect of *At* on LPS-induced NO production, RAW 264.7 cells were co-treated with LPS and scalar amounts of *At*. Then, nitrite production was evaluated as indicator of NOS2 activity. Results showed that *At* in the range of 1.6–6.4 mg/ml significantly diminished nitrite accumulation, as compared to control cells treated with LPS alone (Figure 1B). Moreover, the secretion of the pro-inflammatory cytokine IL-6 was reduced in culture supernatant of RAW264.7 co-exposed to LPS and *At* (Figure 1C). In particular, within the *At* dose rage effective in countering NOS2 activity, LPS-dependent production of IL-6 was the greater inhibited the higher was *At* concentration, with a significant cytokine decrease at 3.2 and 6.4 mg/ml of *At* (*p* = 0.0014 and 0.0003, respectively). Decreased expression of IL-1β, IL-6, TNFα and IL-23p19 tested by Real-time PCR confirmed the anti-inflammatory effect of *At* (data not shown).

In summary, after having identified an *At* range of doses preserving cell viability and suitable to study *At* anti-inflammatory activity, we demonstrated that *At* water extract can successfully restrain NOS2 activity and reduce the release of pro-inflammatory IL-6 by RAW 264.7 exposed to LPS.

### *At* Extract Inhibits RANKL-Induced Osteoclastogenesis in RAW264.7

**FIGURE 2:**
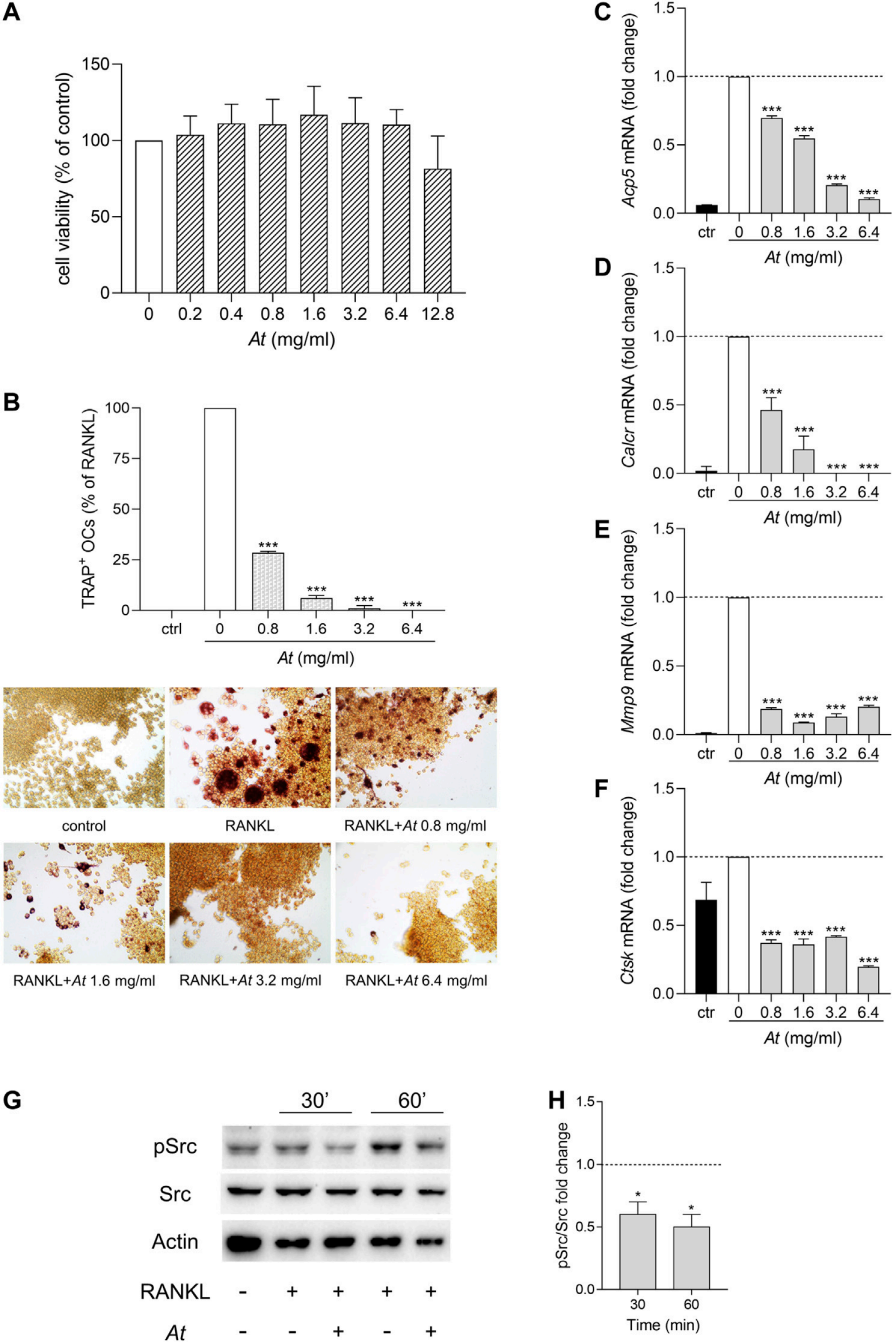
Suppression of osteoclastogenesis by *At* extract in RANKL-differentiated RAW264.7 cells. **(A–F)** RAW264.7 cells were *in vitro* co-treated for 5 days with RANKL and *At* extract at the indicated concentrations; ctr, RAW264.7 cells cultured without RANKL. **(A)** Cell viability was evaluated by MTT assay. The percentage of viable cells respect to RANKL control was reported as the mean of three independent experiments, each conducted in triplicate. **(B)** Pictures in the lower panels: representative staining of TRAP-positive osteoclasts, induced by RANKL from RAW264.7 cells in the presence of the indicated concentrations of *At* extract; upper panel: quantitative analysis of the number of osteoclasts, represented as TRAP-positive cell percent, respect to RAW264.7/RANKL control (white bar). Results are reported as mean ± SD of the osteoclasts number counted in randomly selected visual fields in different areas of each well. ***p < 0.001, *At-treated vs RANKL-treated* cells (one-way ANOVA test). **(C–F)** Real-time PCR analysis of *Acp5, Calcr, Mmp9, Ctsk* transcripts in RAW264.7/RANKL treated with the indicated concentrations of *At* extract; ctr, RAW264.7 cells cultured without RANKL. Gene expressions were normalized to the expression of *Gapdh* and reported as relative to the normalized expression in RAW264.7/RANKL (white bar; dotted line, fold = 1). Data (mean ± SD) are the results of three independent measurements. ***p < 0.001, *At*-treated vs RANKL-treated cells (one-way ANOVA test). **(G,H)** Inhibition of RANKL-induced activity of Src in *At*-pretreated RAW264.7 cells. Expression of the phosphorylated (pSrc) and total (Src) protein level of the kinase Src in RAW264.7 cells pretreated 1 h with 3.2 mg/ml of *At* extract and stimulated with RANKL for 30 and 60 min. Actin expression was used as sample normalizer. One representative immunoblot of three is shown **(G)**. pSrc/Src ratio is calculated by densitometric quantification of the specific bands detected in three independent experiments **(H)**. Data (mean ± SD) are reported as fold change of samples co-treated with *At* and RANKL relative to RANKL-stimulated cells (dotted line, fold = 1). *p < 0.05 (one-way ANOVA test).

Local strong inflammation and progressive destruction of affected synovial joints are characteristics of RA. Bone erosion is mainly caused by the activity of osteoclasts, multinucleated cells originated from monocyte/macrophage lineage by cell fusion. To investigate the anti-osteoclastogenic potential of *At*, we induced RAW264.7 differentiation into osteoclasts by RANKL, in the presence of *At*. In the tested dose range (0.2–12.8 mg/ml), MTT assay revealed no significant effect of *At* on cell viability (Figure 2A). However, since the highest concentration of *At* displayed a decline, a narrower range of *At* doses (0.8–6.4 mg/ml) was chosen to assess *At* ability in inhibiting osteoclast differentiation process. After 5-days culture, cells were processed for tartrate-resistant acid phosphatase (TRAP) staining to visualize TRAP-positive purple-pink multinucleated osteoclasts (Figure 2B, lower panels). The inhibitory effect of *At* on RANKL-induced osteoclast differentiation in RAW 264.7 cells was evaluated by counting the number of TRAP-positive cells having three or more nuclei. Results showed that RANKL-dependent osteoclast formation was strongly inhibited by *At* treatment (Figure 2B, upper panel). Indeed, the percentage of TRAP-positive cells was significantly reduced by *At* (*p* < 0.0001 at all of the tested concentrations), reaching the complete inhibition of RANKL-induced osteoclastogenesis in samples stimulated with 6.4 mg/ml of *At* (Figure 2B). The decreased TRAP enzymatic activity reflected the down-modulation of *Acp5* gene, encoding for TRAP and considered a marker of osteoclast function (Minkin, 1982) (Figure 2C). To further confirm the effect of *At* on RANKL-induced osteoclastogenesis, we measured the expression level of several markers of osteoclast formation and function, including calcitonin receptor (*Calcr*), matrix metallopeptidase 9 (*Mmp9*) and cathepsin K (*Ctsk*). Real-time PCR analysis demonstrated that *At* significantly (*p* < 0.0001) and in a dose dependent manner reduced the expression of *Calcr* (a gene highly expressed in mature osteoclasts (Roodman, 1996)), *Mmp9* (encoding for a protein belonging to a family of extracellular matrix-degrading enzymes involved in tissue remodeling) and *Ctsk* (a cysteine protease highly expressed in osteoclasts and involved in collagen degradation) respect to RANKL control (fold change assigned value = 1) (Figures 2D–F).

The non-receptor tyrosine kinase Src plays an essential role in actin dynamics and its organization in osteoclasts (Destaing et al., 2008). Indeed, Src deficient mice develop a condition known as osteopetrosis caused by a defect of bone resorption (Soriano et al., 1991). Upon RANKL binding, RANK receptor recruits Src and promotes the nuclear translocation of NFATc1, an important transcription factor that regulates the expression of osteoclastogenesis-related genes (Zhi et al., 2020). On analyzing the effect of *At* on RANK signaling pathway, we investigated whether *At* could modulate the RANKL-induced activity of Src. Therefore, RAW 264.7 macrophages were pre-treated with *At* (3.2 mg/ml) for 1 h and then exposed to RANKL for additional 30 and 60 min. The analysis of phosphorylated Src at the tyrosine 424 in the activation loop (Manni et al., 2020) was evaluated by western blotting experiments as an indicator of kinase activation (Barker et al., 1995; Bessede et al., 2014; Roskoski, 2015; Volpi et al., 2016; Mondanelli et al., 2017b). Results demonstrated that RANKL promoted Src activation in RAW264.7 macrophages, an effect that was abrogated in the presence of *At* (Figure 2G,H). Nevertheless, Src phosphorylation was significantly decreased also in RANKL-induced osteoclasts after 90 min of stimulation with RANKL, respect to control not pretreated with *At* (Supplementary Figure S2).

Therefore, our data demonstrated that *At* water extract can effectively interfere with RANKL-induced osteoclastogenesis by altering RANK signaling pathway and thus reducing the expression of key genes responsible for osteoclast formation and function.

### Effect of *At* on CIA Progression

**FIGURE 3:**
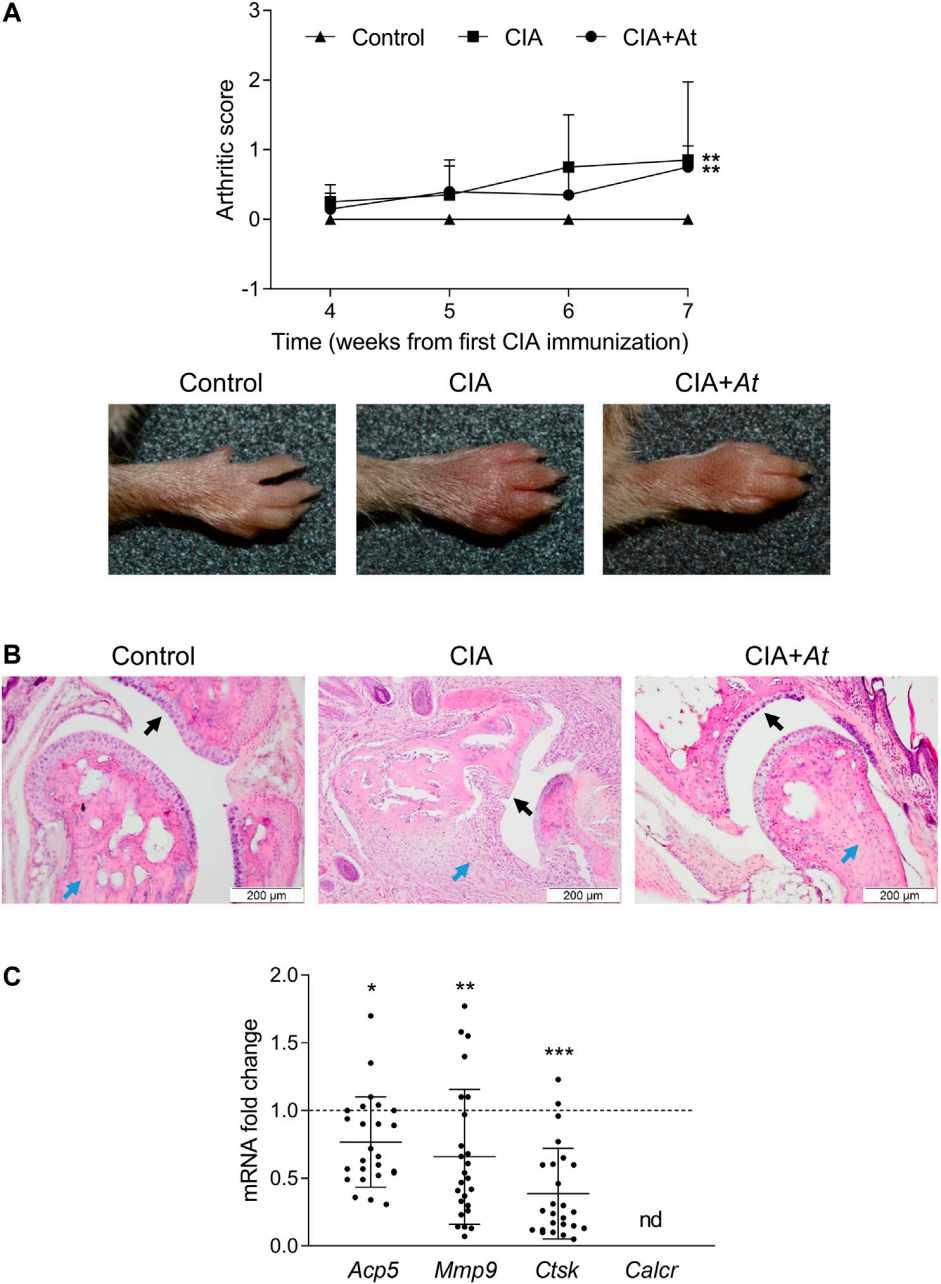
Effect of *At* decoction on joints of CIA-induced mice. **(A)** Mean arthritis score of all the limbs for each group (upper panel) and representative paw appearance (lower panels) following the second collagen immunization in groups of mice not CIA-immunized and drinking water (Control), CIA-immunized and drinking water (CIA), CIA-immunized and drinking *At* decoction (CIA+*At*). **(B)** Histological assessment of joint tissue from a representative mouse in each group. Black and blue arrows indicate cartilage and inflammatory infiltrate, respectively, in the joints of mice in groups not CIA-immunized and drinking water (Control), CIA-immunized and drinking water (CIA), CIA-immunized and drinking *At* decoction (CIA+*At*). Data are presented as mean values ± SD. Statistical analysis was performed with the one-way ANOVA test: **p < 0.01, CIA and CIA+*At* vs Control. **(C)** Real-time PCR analysis of *Acp5*, *Mmp9*, *Ctsk* and *Calcr* transcripts in joint tissue sample from CIA+*At* group. Gene expressions were normalized to the expression of *Gapdh* and reported as relative to the normalized expression in CIA group (dotted line, fold = 1). Data (mean ± SD) are the results of five independent measurements. ***p < 0.001, **p < 0.01, *p < 0.05, CIA+*At* vs CIA (paired Student’s *t* test).

To give insights in *At* effect on bone preservation *in vivo*, we assayed histologic integrity of cartilage and expression of osteoclast marker genes in CIA-induced mice treated with *At* decoction. Mice were classified into the following groups: 1) not immunized, nor treated with *At* (Control) (*n* = 5); 2) immunized to induce CIA, but not treated with *At* (CIA) (*n* = 5); 3) immunized and treated with *At* from the first day of immunization (CIA+*At*) (*n* = 5). Arthritis induction was scored following the criteria previously outlined (Brand et al., 2007; Adorisio et al., 2019). After the second CIA immunization, signs of arthritis incidence started to became visible at week four ranging from score 1 to 2 (in Figure 3A, for each group mean severity score of all the limbs are plotted). Arthritis development became more evident from week 6, with limb arthritic scores ranging up to 4. On week 7, the increase in arthritic score of CIA and CIA+*At* groups were statistically significant, compared to control animals. Concomitantly with the CIA development, clinical signs of suffering (which did not compromise their overall welfare) became visible, probably due to the limb pain, inflammation and swelling. Although mice administrated with drinking *At* showed only a slight, not significant, decrease in the incidence of arthritis development in terms of joint swelling (Figure 3A), nevertheless, in these animals joint histology enlightened *At* protective effect against CIA-dependent bone loss (Figure 3B). In fact, while mice with CIA exhibited altered cartilage with many visible niches and dramatic inflammatory infiltrate, in those receiving *At* water extract cartilage appeared as intact and smooth as in control group, and no signs of inflammatory infiltration were observed. Real-time PCR analysis of osteoclast marker genes on joint tissue samples revealed a significantly decreased expression of *Acp5* (*p* = 0.03), *Mmp9* (*p* = 0.008) and *Ctsk* (*p* < 0.001) in CIA+*At* respect to CIA group, while *Calcr* was not detectable at all. (Figures 3C).

Thus, *in vivo* administration of *At* water extract, though only in part limiting paw edema, was impressively effective in preventing histological signs of CIA at joint level, an effect associated with significant decrease of osteoclast markers.

### Anti-Inflammatory and Anti-osteoclastogenic Properties of TAT-2 Glycoside

**FIGURE 4:**
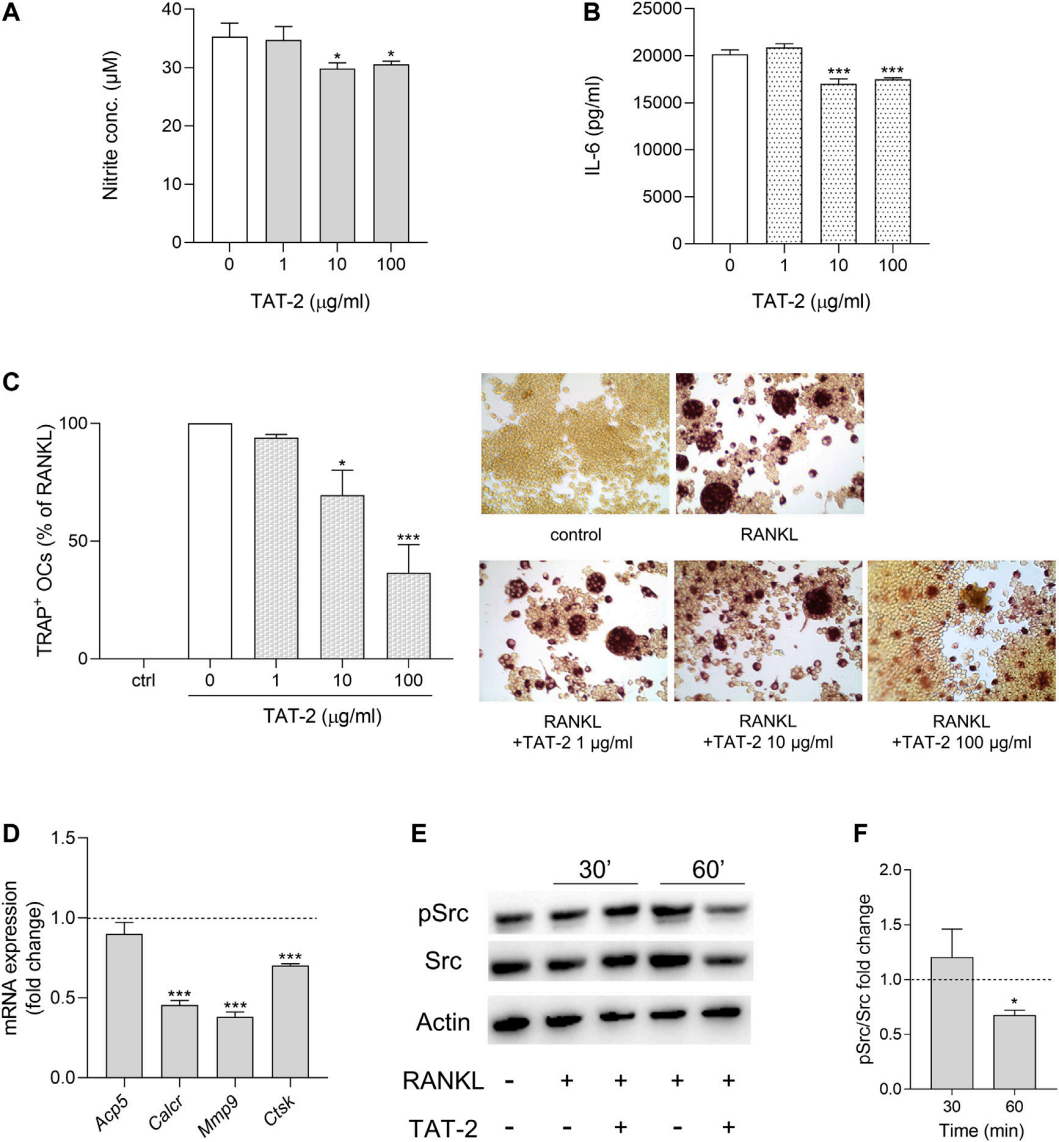
Anti-inflammatory and anti-osteoclastogenic activity of TAT-2 glycoside. **(A,B)** LPS-activated cells were treated for 24 h with TAT-2 glycoside at the indicated concentrations. **(A)** NO release in the culture supernatant of LPS-activated RAW 264.7 cells was quantified as nitrite concentration. **(B)** The concentrations of IL-6 in the supernatants of LPS-activated RAW 264.7 cells *in vitro* stimulated with the indicated concentrations of TAT-2 were determined by ELISA test. Results are reported as mean ± SD of three independent experiments, each conducted in triplicate. *p < 0.05, ***p < 0.001, TAT-2-treated vs LPS-treated cells (one-way ANOVA test). **(C,D)** RAW264.7 cells were *in vitro* co-treated for 5 days with RANKL and TAT-2 glycoside at the indicated concentrations. **(C)** Pictures in the right panels: representative staining of TRAP-positive osteoclasts induced by RANKL from RAW264.7 cells in the presence of the indicated concentrations of TAT-2 glycoside; left panel: quantitative analysis of the number of osteoclasts represented as TRAP-positive cell percent, respect to RAW264.7/RANKL control (white bar); ctr, RAW264.7 cells cultured without RANKL. Results are reported as mean ± SD of the osteoclasts number counted in randomly selected visual fields in different areas of each well. *p < 0.05, ***p < 0.001, TAT-2–treated vs RANKL-treated cells (one-way ANOVA test). **(D)** Real-time PCR analysis of *Acp5*, *Calcr*, *Mmp9*, *Ctsk* transcripts in RAW264.7/RANKL treated with 100 μg/ml of TAT-2 glycoside. Gene expressions were normalized to the expression of *Gapdh* and reported as relative to the normalized expression in RAW264.7/RANKL (dotted line, fold = 1). Data (mean ± SD) are the results of three independent measurements. ***p < 0.001, TAT-2–treated vs RANKL-treated cells (paired Student’s *t* test). **(E,F)** Inhibition of RANKL-induced activity of Src in TAT-2–pretreated RAW264.7 cells. Expression of the phosphorylated (pSrc) and total (Src) protein level of the kinase Src in RAW264.7 cells pretreated 1 h with 100 μg/ml of TAT-2 and stimulated with RANKL for 30 and 60 min. Actin expression was used as sample normalizer. One representative immunoblot of three is shown **(E)**. pSrc/Src ratio is calculated by densitometric quantification of the specific bands detected in three independent experiments **(F)**. Data (mean ± SD) are reported as fold change of samples co-treated with TAT-2 and RANKL relative to RANKL-stimulated cells (dotted line, fold = 1). *p < 0.05 (one-way ANOVA test).

Maesopsin 4-O-β-D-glucoside (TAT-2) is one of the two most abundant components dosed in *At* water extract (Adorisio et al., 2019; Supplementary Figure S1). By decreasing *Il17* and *Rorc* expression in CD4^+^ T lymphocytes, TAT-2 likely contributes to the reduced Th17 differentiation observed as major effect of *At* on T cells (Adorisio et al., 2019). In the present study, we investigated whether *At* action targeting macrophages might, at least in part, be ascribed to TAT-2. To this purpose, we used the same inflammatory (RAW264.7/LPS) and osteoclastogenic (RAW264.7/RANKL) *in vitro* models already experimented with *At* water extract to assess TAT-2 ability in restraining inflammation and osteoclast differentiation. One, 10 and 100 μg/ml doses, selected according to the previous study (Adorisio et al., 2019), resulted in the absence of negative effects on RAW264.7 cell viability (Supplementary Figures S3, S4). In the inflammatory model, TAT-2 displayed a significant, although not dose dependent, effect in reducing nitrite accumulation (*p* = 0.0112 and 0.0234 at 10 and 100 μg/ml, respectively) (Figure 4A) and IL-6 production (*p* < 0.001 at both 10 and 100 μg/ml) (Figure 4B) by LPS-exposed RAW264.7 cells. In the osteoclast differentiation model, the RANKL-mediated differentiation of macrophages into osteoclasts was clearly inhibited by TAT-2 in a dose-dependent manner, with significant drop of multinucleated TRAP-positive cells as compared to RANKL control (70% ± 11% and 37% ± 12% of reduction, *p* = 0.0236 and 0.0009, in samples treated with 10 and 100 μg/ml of TAT-2, respectively) (Figure 4C). Expression of osteoclast differentiation genes was analyzed in RAW264.7/RANKL cells cultured with TAT-2 at the highest tested concentration (i.e., 100 μg/ml). For each gene, the fold change was calculated respect to RANKL control. All the investigated genes (*Acp5*, *Calcr*, *Mmp9* and *Ctsk*) were down modulated by TAT-2 treatment (Figure 4D). In particular, the down-regulation reached statistical significance (*p* < 0.001) for *Calcr*, *Mmp9* and *Ctsk*, while *Acp5* expression was weakly decreased, in accordance with the residual staining observed in TRAP-positive cells with less than three nuclei, not computable as osteoclasts (multinucleated cells with more than three nuclei). On analyzing Src activation, cell pretreatment with 100 μg/ml of TAT-2 reduced Src phosphorylation at 1 h after RANKL addition, respect to the basal level of phosphorylation induced by RANKL alone (Figures 4E,F).

In summary, negative modulation of NOS2 and IL-6 inflammatory markers, and inhibition of osteoclastogenic activity displayed by TAT-2 in RAW264.7 cells confirmed this glycoside as a bioactive component importantly contributing to effectiveness of *At* decoction not only on T, as previously found (Adorisio et al., 2019), but also on macrophage cell population.

## Discussion

Leaves of *A. tonkinensis* Vietnamese tree are used in traditional medicine to treat autoimmune diseases as rheumatoid arthritis, apparently with no side effect. Scientific validation of the therapeutic activity of leaf extract are documented in several studies. By n-butanol extract, Sung and co-workers of Vietnam Academy of Science and Technology (VAST) in Hanoi, Vietnam, isolated auronol glycosides maesopsin 4-O-β-D-glucoside and alphitonin-4-O-β-D-glucoside endowed with immunosuppressive properties (Thuy et al., 2004). Two other compounds, artonkin-4’-O-glucoside and kaempferol-3-O-β-D-glucoside were found to cause anti-inflammatory effect with different potencies, by suppressing T cell proliferation and cytokine expression in a model of arthritis (Dang et al., 2009). Moreover, maesopsin 4-O-β-D-glucoside (TAT-2) was found to have an antiproliferative effect on activated T lymphocytes (Thuy et al., 2004) and the ability to significantly decrease cell growth of OCI-AML, an acute myeloid leukemia cell line (Pozzesi et al., 2011).

Decoction obtained from *A. tonkinensis* leaves—similar to the Vietnamese traditional medicine preparation—demonstrated a protective effect against arthritis when administered as drinking water to CIA mice, significantly alleviating signs and symptoms of arthritis (Adorisio et al., 2019). This study enlightened *A. tonkinensis* effect on T cell population, revealing Th17 inhibition in joint lymph nodes by gene expression profile and Th17 decreased polarization from splenic CD4^+^ T cells by *ex vivo* analysis. Since macrophages, important actors of RA development, were not yet investigated as cell target of *A. tonkinensis* bioactive compounds, we begun to study the effects of *A. tonkinensis* decoction on macrophage cell type. Monocytes/macrophages have multifaceted roles in RA pathogenesis (Udalova et al., 2016). They are potent producers of cytokines (TNFα, IL-1β, IL-6, IL-10, M-CSF and others) and chemokines (CCL3, CCL5, CX3CL1, CCL2 and CXCL8), can act as professional APCs presenting arthritogenic antigens to the TCR of autoreactive T cells, and considerably contribute to joint destruction (Tran et al., 2007). Moreover, macrophages activate synovium fibroblasts to proliferate and further secrete pro-inflammatory mediators in a positive feedback loop leading to macrophage polarization and accumulation of T and B cells in the synovium. In this context, RA fibroblasts, secreting large amounts of RANKL and M-CSF, promote the formation of osteoclasts and subsequent pathologic bone resorption (Takayanagi et al., 2000). The bone damage is operated at osteoclast mature stage, when cells form an actin ring at the site of bone contact aiding attachment, secrete tartrate-resistant acid phosphatase (TRAP) to destroy the bone matrix and release metalloproteinases (MMP-9) and cathepsin K to degrade bone by removing bone-lining collagen (Visagie et al., 2015). In light of these cell mechanisms, macrophages represent useful therapeutic early targets to restrain subsequent pathogenic cell activation, including that of Th17, which infiltrate synovial tissue of RA patients and produce IL-17, a RANKL-inducer cytokine (Kotake et al., 1999; Nakae et al., 2003).

Our study was focused on *in vitro* models of inflammatory and osteoclastogenic microenvironment, two peculiar RA conditions, reproduced by RAW264.7/LPS and RAW264.7/RANKL cultures, respectively. *A. tonkinensis* decoction, usable in a wide range of concentrations not affecting cell viability, gave a good and dose dependent reduction of LPS-triggered NO production, likely due to *Nos2* down-modulation, rather than enzyme inhibition, as found by real-time PCR analysis (data not shown). Such negative regulation of *Nos2* might be due to the reduction of proinflammatory cytokines, resulting from *A. tonkinensis* treatment. In fact, beside the decreased expression of proinflammatory cytokines IL-1β, TNFα and IL-23p19 (data not shown), IL-6, one of the main markers of pro-inflammatory macrophage response, powerfully induced by LPS in these cells, was well countered by *A. tonkinensis* in a dose dependent manner. This evidence is particularly notable considering the relevance of IL-6 in AR pathogenesis (so that it is therapeutically targeted) (Ohsugi, 2020), and its role in fueling the positive feedback loop leading to Th17 differentiation (Wu and Wan, 2020). However, the most significant effect of *A. tonkinensis* water extract on macrophage cells was at osteoclast formation level, since RANKL-dependent cell fusion into osteoclasts was completely abrogated at the highest tested dose of decoction and the expression of osteoclast functional markers (TRAP, matrix metalloproteinase-9, calcitonin receptor and cathepsin K) importantly dropped respect to RANKL control. The finding is consistent with the hypothesis that bioactive compounds present in *A. tonkinensis* decoction might interfere in the signaling of RANK, the receptor binding RANKL and controlling osteoclast differentiation process. Actually, in this study we have demonstrated that phosphorylation of Src, the upstream kinase in RANK signaling pathway (Zhi et al., 2020), was decreased when RAW264.7 cells were pretreated with *A. tonkinensis* prior to RANKL addition. Reduced protein expression of RANK is another consequence of RAW264.7 treatment with *A. tonkinensis* (data not shown), suggesting an impaired macrophage sensitivity to RANKL osteoclastogenic stimulation. *In vivo* experiment with CIA-induced mice treated with *A. tonkinensis* decoction as drinking water, the same administration route used in traditional medicine, importantly supports *in vitro* data on *A. tonkinensis* interference in osteoclast formation. Though paw edema was weakly limited by the decoction, bone and cartilage integrity largely benefited from *A. tonkinensis* treatment, likely thanks to the strong reduction of osteoclast differentiation and activity (as demonstrated by osteoclast marker gene expression in joint tissue) otherwise observed in CIA control group.

TAT-2 is one of the major component of *A. tonkinensis* water extract, already demonstrated to be active in decreasing *Il17* and *Rorc* gene expression in CD4^+^ T lymphocytes cultured in conditions inducing Th17 differentiation (Adorisio et al., 2019). In the two *in vitro* models assessed in the present study, TAT-2 displayed anti-inflammatory effect (reduced NO and IL-6 production) and anti-osteoclastogenic activity, although weaker respect to those observed by decoction treatment. Since TAT-2 amount contained in the dose of decoction giving the complete abrogation of osteoclast formation is lower (about 15 µg/ml) than the highest TAT-2 tested dose (100 µg/ml), we deduce that TAT-2 is an important, but not the only, compound contributing to the inhibitory effect exerted by *A. tonkinensis* decoction on RAW264.7 macrophages. The leaf extract is likely more effective than TAT-2 thanks to the co-presence of multiple and possibly synergizing bioactive molecules.

In conclusion, our findings that *A. tonkinensis* water extract can restrain macrophage-dependent inflammation and can effectively contrast osteoclast formation and function provide scientific basis to the anti-arthritic efficacy of *A. tonkinensis* decoction, used as traditional medicine remedy. These promising indications may pave the way toward the identification of *A. tonkinensis* new bioactive compounds capable to interfere in the formation of osteoclasts, the cells mainly responsible for joint and bone damage in RA.

## Data Availability Statement

The raw data supporting the conclusion of this article will be made available by the authors, without undue reservation, to any qualified researcher.

## Ethics Statement

The animal study was reviewed and approved by Ethical Commission of the University of Perugia and the Italian Ministry of Health.

## Author Contributions

EO, GM performed experiments and conducted the research; MB designed and supervised the study as a whole; DD, SA prepared and provided *At* decoction, and supervised *in vivo* experiments; MC performed the histology assessment. TT harvested, stored, dried plant leaves, and isolated TAT-2; CO, CV designed individual experiments; MB and GM co-wrote the manuscript. All authors reviewed results and approved the final version of the manuscript.

## Funding

This work was supported by the Italian Ministry of Education, Universities and Research (PRIN2017-2017BA9LM5 to CO; PRIN2017-20173EAZ2Z to CV), by Vietnam Academy of Science and Technology (project VAST.04.08/20-21 to TTT) and by “Fondo Ricerca di Base 2019, Università degli Studi di Perugia” (project entitled “Studio sul processo di preparazione e validazione degli effetti biologici di sostanze naturali selezionate della medicina tradizionale Vietnamita” to DVD).

## Conflict of Interest

The authors declare that the research was conducted in the absence of any commercial or financial relationships that could be construed as a potential conflict of interest.
