# Supplementary figures and images for: Artocarpus tonkinensis Extract Inhibits LPS-Triggered Inflammation Markers and Suppresses RANKL-Induced Osteoclastogenesis in RAW264.7
Source: Front Pharmacol. 2021 Jan 22;11:593829. doi: 10.3389/fphar.2020.593829 (PMC7862131; doi:10.3389/fphar.2020.593829)

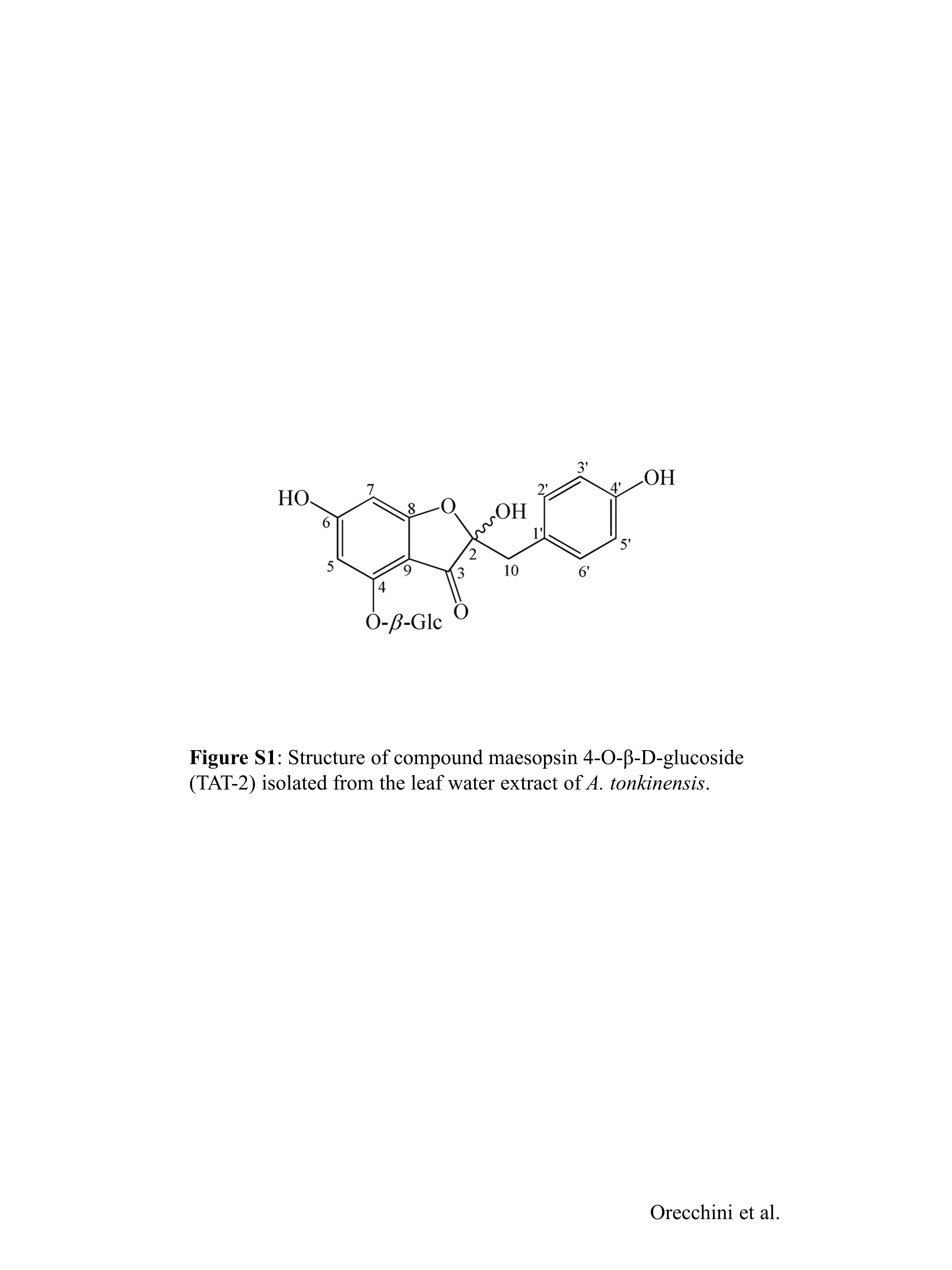

Supplement: Supplementary file 1 [file image1.jpeg]

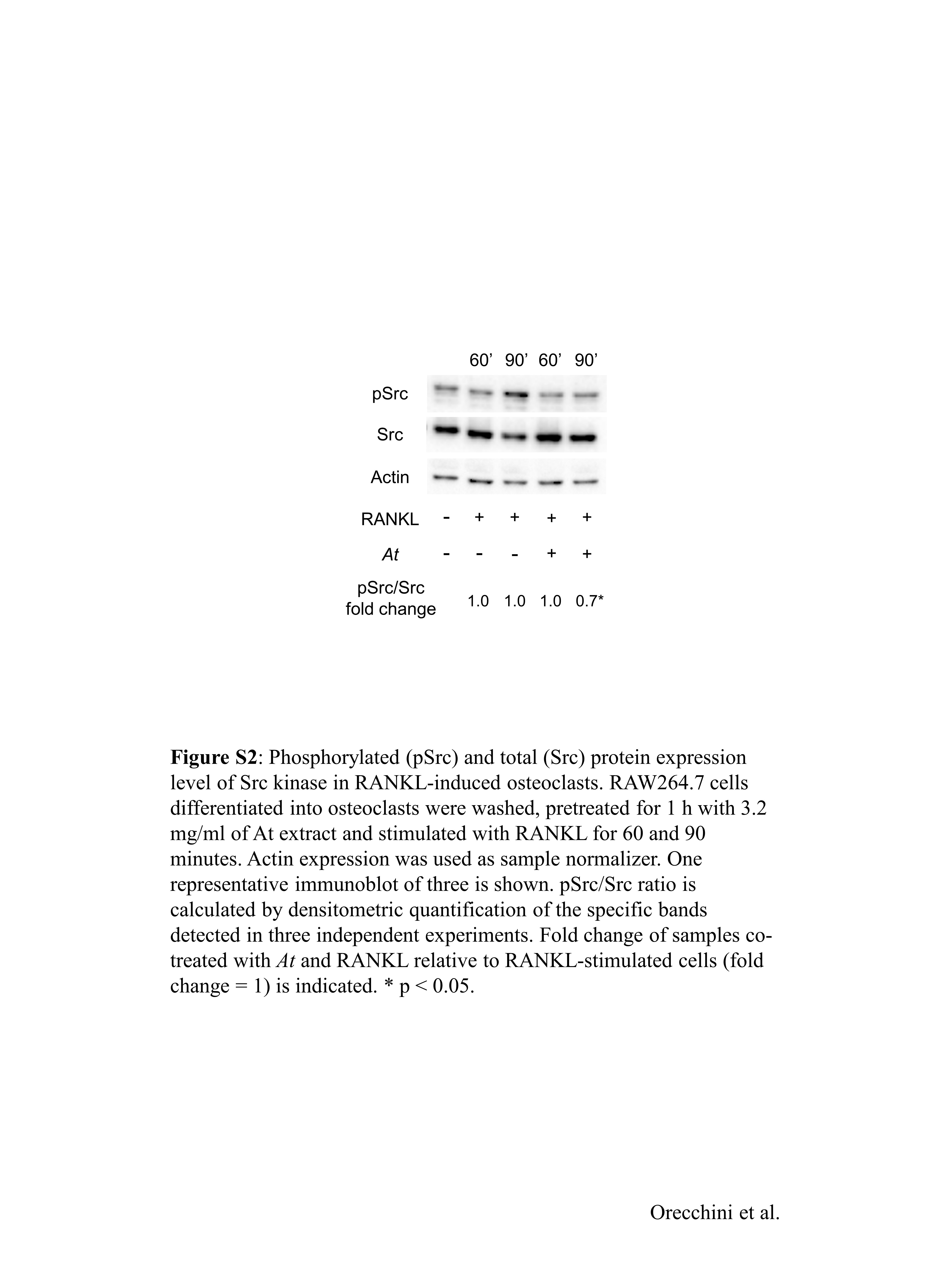

Supplement: Supplementary file 2 [file image2.jpeg]

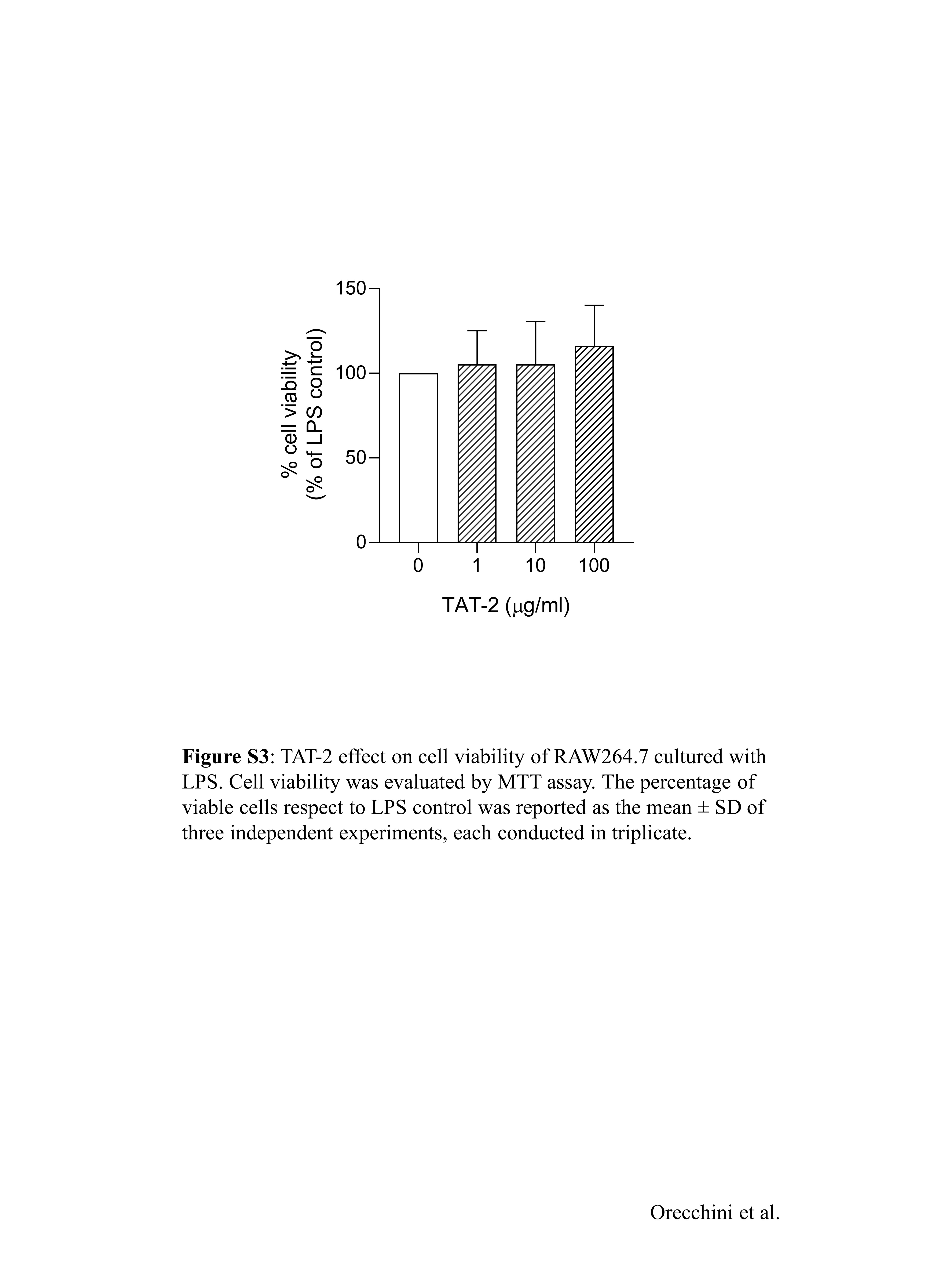

Supplement: Supplementary file 3 [file image3.jpeg]

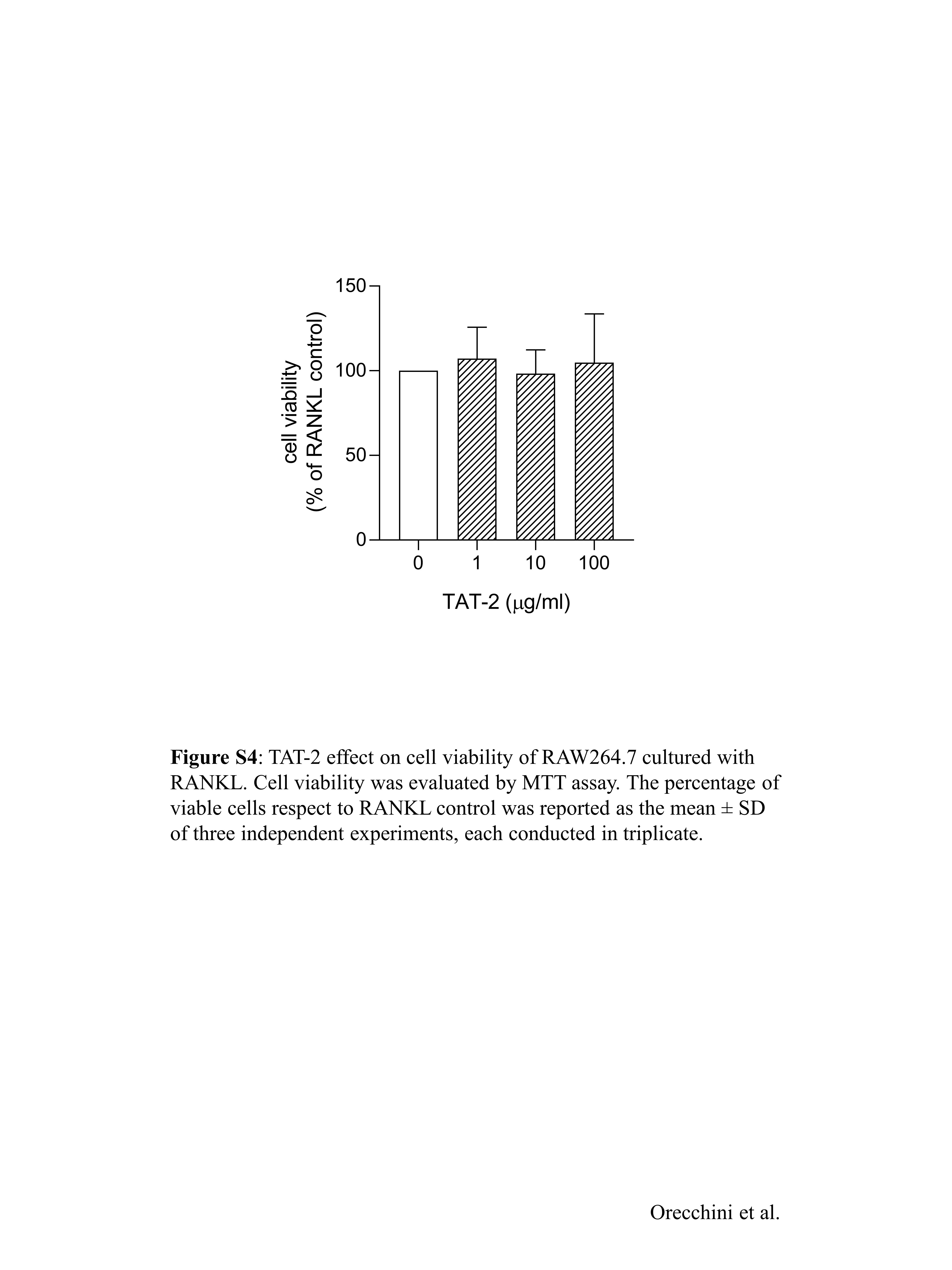

Supplement: Supplementary file 4 [file image4.jpeg]
